# Molecular Fingerprints of Iron Parameters among a Population-Based Sample

**DOI:** 10.3390/nu10111800

**Published:** 2018-11-19

**Authors:** Anne Kaul, Annette Masuch, Kathrin Budde, Gabi Kastenmüller, Anna Artati, Jerzy Adamski, Henry Völzke, Matthias Nauck, Nele Friedrich, Maik Pietzner

**Affiliations:** 1 Institute of Clinical Chemistry and Laboratory Medicine, University Medicine Greifswald, 17475 Greifswald, Germany; anne.kaul@gmx.de (A.K.); annette.masuch@uni-greifswald.de (A.M.); kathrin.budde@uni-greifswald.de (K.B.); matthias.nauck@uni-greifswald.de (M.N.); nele.friedrich@uni-greifswald.de (N.F.); 2 German Center for Cardiovascular Disease (DZHK e.V.), partner site Greifswald, 17475 Greifswald, Germany; voelzke@uni-greifswald.de; 3 Institute of Bioinformatics and Systems Biology, Helmholtz Zentrum München, 85764 Neuherberg, Germany; g.kastenmueller@helmholtz-muenchen.de; 4 Institute of Experimental Genetics, Genome Analysis Center, Helmholtz Zentrum München, 85764 Neuherberg, Germany; anna.artati@helmholtz-muenchen.de (A.A.); adamski@helmholtz-muenchen.de (J.A.); 5 Lehrstuhl für Experimentelle Genetik, Technische Universität München, 85354 Freising-Weihenstephan, Germany; 6 DZD (German Center for Diabetes Research), 85764 München-Neuherberg, Germany; 7 Institute for Community Medicine, University Medicine Greifswald, 17475 Greifswald, Germany; 8 DZD (German Center for Diabetes Research), site Greifswald, 17475 Greifswald, Germany

**Keywords:** iron surrogates, metabolomics, lipoprotein profiling

## Abstract

Iron deficiency is the most frequent deficiency disease and parameters of iron metabolism appear to be linked to major metabolic and cardiovascular diseases. We screened a large set of small molecules in plasma for associations with iron status among apparently healthy subjects to elucidate subclinical profiles which may provide a link between iron status and onset of diseases. Based on mass spectrometry and nuclear magnetic resonance spectroscopy we determined 613 plasma metabolites and lipoprotein subfractions among 820 apparently healthy individuals. Associations between ferritin, transferrin, haemoglobin and myoglobin and metabolite levels were tested by sex-specific linear regression analyses controlling for common confounders. Far more significant associations in women (82 out of 102) compared to men became obvious. The majority of the metabolites associated with serum ferritin and haemoglobin in women comprising fatty acid species, branched-chain amino acid catabolites and catabolites of heme. The latter was also obvious among men. Positive associations between serum transferrin and VLDL and IDL particle measures seen in women were observed in men with respect to serum ferritin. We observed a sexual-dimorphic fingerprint of surrogates of iron metabolism which may provide a link for the associations between those parameters and major metabolic and cardiovascular disease.

## 1. Introduction

Iron is an important co-factor of heme-containing enzymes and proteins. It is the most important transport and storage medium for oxygen and found in descending order in haemoglobin, myoglobin, ferritin and transferrin [1]. Transferrin is the major transport protein whereas ferritin represents the physiological storage, in particular in the liver [2,3]. As serum iron levels highly fluctuate, for example, due to the acute nutritional or inflammatory state [4], serum ferritin, transferrin and haemoglobin are the most useful clinical markers for iron homeostasis. 

Iron deficiency is the world’s most frequent deficiency disease with a highly sex-dependent prevalence—women are significantly more affected than men [5]. However, less is known about the metabolic consequences of alterations in iron metabolism. Previous studies [6,7] suggested surrogates of iron metabolism, for example, ferritin, as markers of incident type 2 diabetes mellitus (T2DM) and disturbances of iron metabolism manifested as anaemia were associated with T2DM. Further, an increase in serum ferritin was associated with increased oxidation of lipoprotein particles among subjects from the general population [8,9]. Even potential relations to cardiovascular disease [10,11,12] or hepatic steatosis [13] were suggested. In conclusion, iron metabolism seems to be linked to whole body metabolism through various paths but a comprehensive characterization of metabolic alterations linked to iron metabolism in humans is still lacking.

To address this issue the investigation of the metabolome, as the entirety of small molecules, by means of mass spectrometry (MS) or ^1^H-nuclear magnetic resonance (NMR) spectroscopy offers the possibility to analyse metabolic processes at the molecular level even in an epidemiological setting [14]. The blood metabolome covers not only (by)products of endogenous metabolism but even effects from lifestyle, medical treatment or the environment. For instance, one previous study [15] showed that the association between red meat consumption and incident T2DM was mediated to a great amount by serum ferritin and several plasma metabolites. Moreover, a beneficial effect of micronutrients supplementation, including iron, is still under debate and hence non-targeted OMICs studies, including metabolomics, were recommended to derive reliable surrogate markers [16]. 

In summary, the comprehensive metabolic characterization of alterations in iron metabolism could fulfil two aims: (1) the identification of metabolic links to iron homeostasis associated disease and (2) naming of potential biomarkers of adequate iron availability. To this end, we associated clinically relevant markers of iron metabolism (haemoglobin, myoglobin, ferritin and transferrin) with targeted and untargeted metabolomics data, derived by MS and ^1^H-NMR, from a population-based sample comprising more than 800 individuals.

## 2. Materials and Methods

### 2.1. Study Population

The Study of Health in Pomerania (SHIP-TREND) is a population-based study conducted in West Pomerania, a rural region in north-east Germany and a detailed description of the sampling procedure and the study population can be found elsewhere [17]. In total, 4420 subjects chose to participate (50.1% response). All participants gave written informed consent before taking part in the study. The study was approved by the ethics committee of the University of Greifswald and conformed to the principles of the declaration of Helsinki. SHIP data are publicly available for scientific and quality control purposes by application at www.community-medicine.de.

For a subsample of 1000 subjects without self-reported diabetes plasma and urine metabolomics data based on MS and ^1^H-NMR were available. Subjects exhibiting at least one of the following criteria were excluded: (1) missing values in exposure or confounding variables (*N* = 54), (2) intake of iron modifying medication (*N* = 4; ATC code B03AA), (3) acute inflammation (*N* = 28; hsCRP > 10 mg/L), (4) impaired renal function (*N* = 7; eGFR < 60 mL/min/1.72 m²), or (5) use of contraceptives (*N* = 87; ATC code G03A and G02B). In total, 820 subjects were available for subsequent analyses.

### 2.2. Laboratory Measurements and Phenotypic Characterization

Smoking status (current, former or never smokers), daily alcohol consumption and physical activity (≥ 1 h training a week) were assessed using computer-aided personal interviews. Waist circumference (WC) was measured to the nearest 0.1 cm using an inelastic tape midway between the lower rib margin and the iliac crest in the horizontal plane. Menopausal state of the women was categorized using a previously published procedure [18]. Briefly, women not older than 40 years or not older than 60 years but reporting menstrual cycle were classified as premenopausal (*N* = 150). Fasting blood samples were taken from the cubital vein of participants in the supine position between 7.00 a.m. and 13.00 p.m. In the same time span spot urine samples were taken. All samples were either analysed immediately or stored at −80 °C. Serum concentrations of ferritin were measured using an immunoassay (Dimension VISTA, Siemens Healthcare Diagnostics, Eschborn, Germany), with coefficient of variation of 4.9%, 2.7% and 2.6% at low, medium and high concentrations, respectively. Serum concentrations of transferrin were measured using a nephelometric assay (Dimension VISTA, Siemens Healthcare Diagnostics, Eschborn, Germany) with a coefficient of variation of 3.5% and 2.9% at low and high concentrations, respectively. Serum concentrations of myoglobin were measured using an enzyme immunoassay (Dimension VISTA, Siemens Healthcare Diagnostics, Eschborn, Germany) with a coefficient of variation of 2.1% and 2.8% at low and high concentrations, respectively. Haemoglobin concentrations were analysed in EDTA whole blood samples using the Sysmex XN-9000 Haematology Autoanalyzer (Sysmex, Kobe, Japan) according to the manufacturer’s recommendation. Serum cystatin C concentrations were measured using a nephelometric assay (Dimension VISTA, Siemens Healthcare Diagnostics, Eschborn, Germany) and subsequently the estimated glomerular filtration rate (eGFR) was calculated using the CKD-EPI equation [19]

### 2.3. Metabolomics Measurements

A detailed description of all applied measurement techniques has been published before [20,21]. Briefly, three different approaches were combined: (1) non-targeted MS-based profiling of plasma samples as reported previously [22], (2) targeted MS-based profiling of plasma samples using the AbsoluteIDQ p180 Kit (BIOCRATES LifeSciences AG, Innsbruck, Austria) and (3) ^1^H-NMR-based profiling of plasma samples to derive lipoprotein particles.

After quality control and pre-processing 613 plasma metabolites were available for statistical analyses. Phospholipids obtained with the AbsoluteIDQ p180 Kit follow a specific nomenclature: PC—phosphatidylcholine, lysoPC—lysophoshatidylcholine; SM—sphingomyelin; PC aa/ae—diacyl/ether PC and CXX:Y is the sum of carbon atoms of the side chain(s) followed by the number of doubled bounds. Note that, 177 plasma metabolites could not be unambiguously assigned to a chemical identity and are referred to hereafter with the notation “X” followed by a unique number. Data on lipoprotein particles comprise 117 measures describing the gradient from very-low-density lipoprotein (VLDL) particles to high-density lipoprotein (HDL) particles, including their triglycerides, cholesterol, free cholesterol, phospholipid as well as apolipoprotein B (ApoB), A1 (Apo-A1) and A2 (Apo-A2) content.

### 2.4. Statistical Analysis

Sex-specific linear regression models were created to test for an association between serum ferritin, transferrin, myoglobin and haemoglobin concentrations as independent and metabolites/lipoproteins levels as dependent variables, controlling for age, WC, smoking behaviour, eGFR and serum ALT activities. Ferritin, myoglobin and metabolites levels were log_2_-transformed indicating a clear improvement towards normality. Sex-specific analyses were indicated by numerous significant sex-interaction terms in preliminary statistical exploration. Using a similar approach we tested for a modifying effect of menopause in women. To combine the metabolome data with lipoproteins, linear regression models were run with the lipoprotein as exposure and the metabolite as outcome controlling for age, sex and BMI. To account for multiple testing, we adjusted the *p*-values from regression analyses by controlling the false discovery rate (FDR) at 5% using the Benjamini-Hochberg procedure. Statistical analyses were performed using SAS version 9.4 (SAS statistical software, version 9.4, SAS Institute, Inc; Cary, NC, USA) and R 3.1.1 (R Foundation for statistical computing, version 3.1.1, The R foundation, Vienna, Austria). We investigated possible markers to differentiate high from low, possibly depleted, iron status. To this end, we adapted a previously published workflow [23] to derive a small, namely ten, metabolites containing signature to discriminate between both states. Since cut-offs for iron status greatly varies and given our apparently healthy population we chose a pragmatic approach relying on the distribution of all four markers within our population. First, we divided all participants according to sex-specific quartiles of each marker and subsequently summed the belonging to each quartile up to an iron status score (e.g., lowest ferritin quartile equals 1 and highest quartile equals 4). Based on this score we categorized all participants with a score less than or equal to 7 as being iron depleted and all participants with a score above 13 as being iron rich/sufficient. This approach resulted in groups of approximately equal sizes (*N* = 59/50 men and *N* = 62/47 women). For classification purpose we used a random-forest approach embedded in a two-stage cross-validation procedure allowing for variable selection and robustness testing.

## 3. Results

Characteristics of the study population are displayed in Table 1. Women were less often smoker and had a lower WC, eGFR as well as serum ALT activities. Only hsCRP concentrations were higher among women compared to men. With the exception of transferrin all markers of iron metabolism were significantly lower in women compared to men. 

### 3.1. Surrogates of Iron Metabolism and the Plasma Metabolome

**Figure 1:**
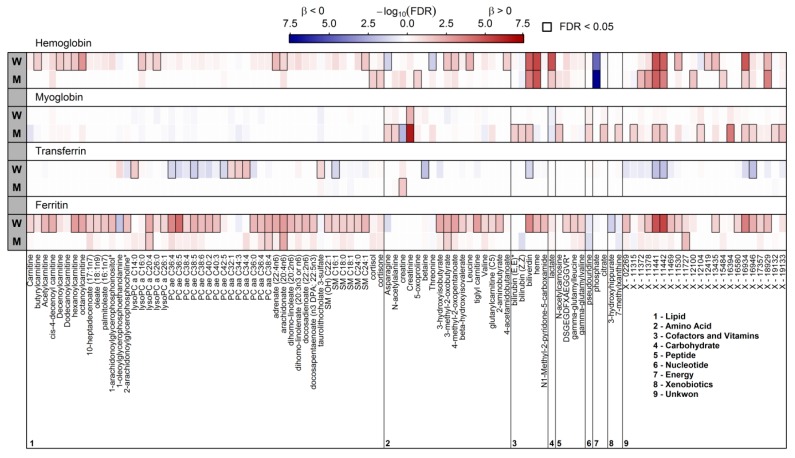
Heatmap of corrected *p*-values (controlling the false discovery rate (FDR) at 5%) from sex-specific (M—men; W—women) linear regression analyses using ferritin, transferrin, myoglobin and haemoglobin concentrations as exposure and plasma metabolites as outcome. Models were adjusted for age, waist circumference, smoking behaviour, estimated glomerular filtration rate and serum alanine aminotransferase activity. Orange and blue shadings indicate positive and inverse associations, respectively. Thick frames indicate significant (FDR < 0.05) associations. Corresponding estimates and FDR values are given in Table S1.

**Figure 2:**
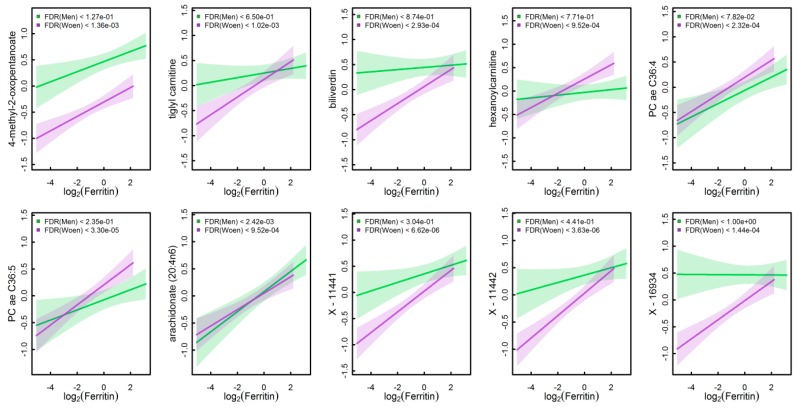
Predicted means and 95%-confidence interval of metabolites levels along serum ferritin concentrations based on linear regression models as outlined in the main text. Effect-estimates were separated by sex (men—green, women—purple). *p*-values after correcting for multiple testing, controlling the false discovery rate at (FDR) 5%, are given in the legend. Metabolite levels are given on a standardized scale where zero represents the population average and one refers to a shift of one standard deviation.

In total, 102 plasma metabolites significantly associated with at least one of the iron parameters in either men or women (Figure 1 and Table S1). No metabolite was common to all four markers under investigation. Strongest associations were observed for serum ferritin. Given the strong sexual dimorphic associations we present results for men and women separately. In addition, a graphical display of the estimated effect of serum ferritin on the ten metabolites showing the strongest associations in both men and women is given in Figure 2.

### 3.2. Significantly Associated Plasma Metabolites in Women

In general, associations seen in the plasma metabolome were much more pronounced in women compared to men, that is, 82 out 102 metabolites associated in total were seen in women only. Ferritin and haemoglobin were the strongest traits with 62 and 27 significantly associated metabolites, respectively. The overlap between both comprised 12 positively associated metabolites, including lipid species, for example, arachidonate or hexanoylcarnitine, metabolites related to heme metabolism, for example, biliverdin (also shared with transferrin) and several unknown compounds (Figure 1) as well as 3-methyl-2-oxobutyrate and 4-methyl-2-oxopentanoate. Apart from this overlap, ferritin levels were positively associated with a number of lipid species, comprising acylcarnitines, none-esterified fatty acids (NEFAs; for example, saturated, monounsaturated and polyunsaturated (PUFA)), phosphatidylcholines (PCs; for example, PC ae C36:4 or PC ae C40:2) and sphingomyelins (SM; for example, SM (OH) C22:1 or SM C24:1). Further positive associations with ferritin concentrations were observed for intermediates of branched-chain amino acid metabolism (BCAA; for example, leucine or 3-methyl-2-oxobutyrate), piperine, peptides (e.g., gamma-glutamylleucine) as well as several unknown compounds (Figure 1). Inverse associations with ferritin levels were limited to 1-oleoylglycerophosphoinositol and 3-hydroxyhippurate. Unique associations with haemoglobin concentrations comprised levels of the cholesterol metabolite 7-alpha-hydroxy-3-oxo-4-cholestenoate and lactate (positively) as well as phosphate and threonine (inversely). An overlap between associations to ferritin and transferrin levels became obvious with respect to levels of ether PCs (PC ae C16:4 and C38:5) and the SM C16:1 with opposing association directions (inversely with transferrin). Unique associations with serum transferrin were obvious for levels of betaine (inversely), diacyl PCs (e.g., PC aa C34:4), the lysophosphatidylcholine (LysoPC) C14:0 and taurolithocholate 3-sulfate.

Myoglobin concentrations associated positively with levels of creatinine only.

Despite no iron marker—plasma metabolite association was significantly modified by menopausal state after correcting for multiple testing, comparing the β-estimates between pre- and post-menopausal women revealed few ferritin-associated metabolites with differing effects (Figure S1). For instance, the inverse association between serum transferrin and PC ae C42:5 was seen in postmenopausal women only, whereas the associations with serum transferrin and biliverdin derivatives was almost absent among them. Further, the association between serum ferritin and valine for instance was stronger among postmenopausal women as well. However, in general there was a good agreement between the estimates in both groups.

### 3.3. Significantly Associated Plasma Metabolites in Men

As already stated compared to women associations in men were much less pronounced. With respect to ferritin, positive associations with levels of the lysoPC a C20:4, PC aa C38:4, arachidonate, 3-methyl-2-oxobutyrate and the unknown X-11727 became apparent. Only creatine levels were positively associated with transferrin in men, while creatine was inversely associated with myoglobin concentrations. Compared to women associations with myoglobin were more frequent in men. Those included positive associations with levels of asparagine, n-acetylalanine, creatinine, biliverdine derivatives, pseudouridine, citrate, 7-methylxanthine and several unknown compounds. Not at least, with respect to haemoglobin concentrations, associations with levels of biliverdin, heme, lactate and phosphate were similar as in women, whereas positive associations with levels of cortisol/cortisone and glutamine were unique to men.

### 3.4. Surrogates of Iron Metabolism and Lipoprotein Particles

**Figure 3:**
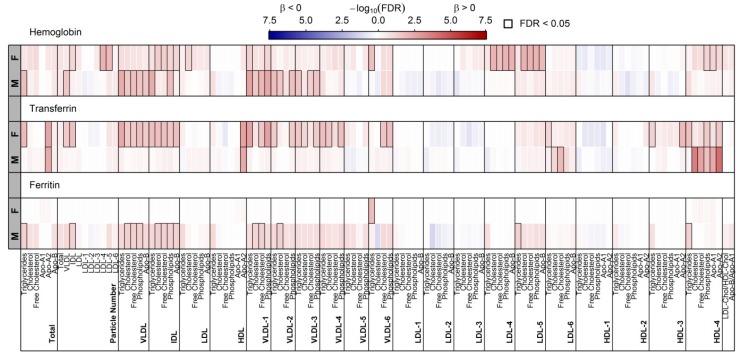
Heatmap of corrected *p*-values (controlling the false discovery rate (FDR) at 5%) from sex-specific (M—male; F—female) linear regression analyses using ferritin, transferrin, myoglobin and haemoglobin concentrations as exposure and lipoprotein particles as outcome. Models were adjusted for age, waist circumference, smoking behaviour, estimated glomerular filtration rate and serum alanine aminotransferase activity. Orange and blue shadings indicate positive and inverse associations, respectively. Thick frames indicate significant (FDR < 0.05) associations. VLDL = very low-density lipoprotein; LDL = low-density lipoprotein; IDL = intermediate-density lipoprotein; HDL = high-density lipoprotein; Apo = apolipoprotein.

Even associations with lipoprotein particles showed in part sexual dimorphic associations (Figure 3). With respect to ferritin concentrations, only the triglyceride (TG) content of small VLDL-particles was positively associated among women whereas in men significant positive associations with total triglycerides (TG), VLDL and IDL particles became apparent. In detail, the positive association with VLDL-particles was mainly due to a positive association with large VLDL particles. Numerous and exclusively positive associations were obvious with transferrin levels in women, comprising total TG and total Apo-A2 levels as well as VLDL, IDL and small-dense HDL particles (HDL_4_). The associations with total Apo-A1 and HDL_4_ were replicated in men. No associations with respect to myoglobin became obvious. Haemoglobin levels showed a divergent association pattern in men and women. Positive associations with small-dense LDL particles (LDL_5_ and LDL_6_) as well as small-dense HDL particles (HDL_4_) were unique to women. In contrast, haemoglobin levels were positively associated with total TG and VLDL particles in men.

### 3.5. Identification of Markers to Differentiate High from Low Iron Status

In an attempt to discriminate between participants with low and high iron markers we achieved moderate discrimination as shown by a median area under the receiver operating curve (AUC) of 0.81 in women and men using a maximum of 10 variables (Figure S2). Validation runs in women were more consistent as shown by less variation in the achieved AUC. Briefly, in women age and eGFR were the most important predictors followed by plasma levels of gamma-glutamylphenylalanine and lysoPC a C16:1. Among men biliverdin, 3-(4-hydroxyphenyl)lactate, gamma-glutamylphenylalanine and waist circumference were the most important variables.

## 4. Discussion

Iron homeostasis is under tight physiological control and monitored in the clinic using circulating surrogates, that is, ferritin, transferrin, haemoglobin and myoglobin. However, perturbations lead to several metabolic adaptations and within the present study we report for the first time a comprehensive panel of small molecules and related alterations in lipoprotein metabolism which were strongly associated with those markers in particular among women. Our results clearly emphasize serum ferritin as the most important marker reflecting numerous metabolic signatures.

### 4.1. Overlapping Metabolites Reflect Heme Degradation

**Figure 4:**
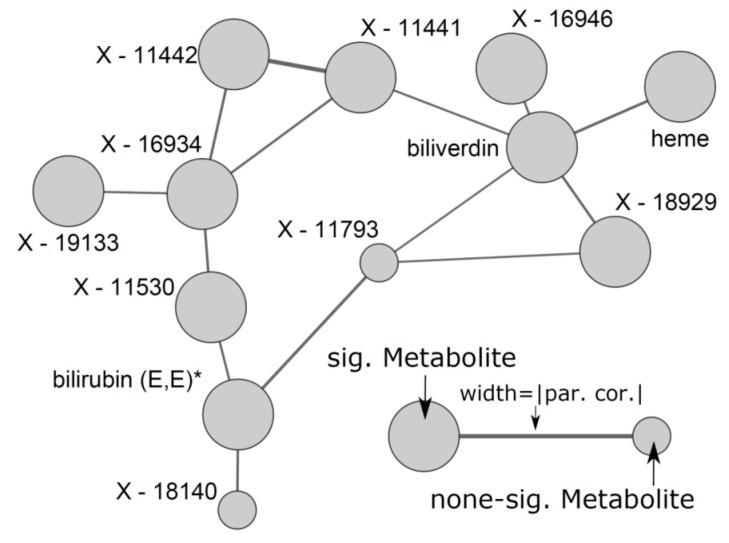
Subnetwork from the Gaussian graphical model to reconstruct metabolite dependencies with a particular focus on heme related metabolites. Increased node size indicates significant associations with at least one red blood cell count trait under investigation. par. cor. = partial correlation.

The strongest associations seen in the present study comprised heme, billiverdin as well as a number of closely related unknown metabolites forming a tight cluster in the estimated GGM (Figure 4). High levels of haemoglobin likely indicate a higher production/turnover of red blood cells and subsequently degradation of heme. The sex-specific association with respect to serum ferritin in women might result from the stronger correlation between haemoglobin and ferritin in women (*r* = 0.35; *p* < 0.01) compared to men (*r* = 0.21; *p* < 0.12), in line with the higher prevalence of iron deficiency in women resulting in both lower ferritin and lower haemoglobin concentrations.

### 4.2. A Complex Metabolic Pattern is Associated with Serum Ferritin

An important finding is given by the interrelation of serum ferritin, plasma NEFAs and the TG-content of small dense VLDL-particles (VLDL_6_) among women. Namely, we observed a positive association between VLDL measures and NEFAs (Figure S3), while serum ferritin was associated to VLDL_6_ TG (Figure 3) as well as to those NEFAs as was observed for VLDL measures (Figure 1 and Figure S3) creating a triangle of association. 

The amount of VLDL particles secreted by the liver is extremely variable. One of the primary determinants is the amount of NEFAs entering the liver. With respect to the origin of NEFAs, the hydrolysis of TG by lipoprotein lipase from VLDL and other TG-rich lipoproteins as well as the lipolysis in adipose tissue by hormone-sensitive lipase play important roles [24]. Of note, in the present study, participants were fasting for at least 8 h by study design. In the fasting condition, NEFAs are primarily released from adipose tissue to fuel muscles [24]. It has been suggested that VLDL particles are smaller and more numerous if saturated fatty acids predominate in the synthesized TG compared to the case if poly-/unsaturated fatty acids are more abundant [25]. NEFAs associated with TG-content of VLDL_6_ were predominantly unsaturated fatty acids. It may be conceivable, that the majority of saturated fatty acids has been incorporated into VLDL particles, thus, with increasing amounts of secreted VLDL_6_ the amount of saturated NEFAs in the circulation decreased. But the underlying molecular mechanisms remain elusive. Nevertheless, the present observations are in line with reported findings considering pathological conditions. For instance, increased NEFA levels were related to insulin resistance [26]. Increased levels of VLDL particles among others are considered also a hallmark of affected lipoprotein metabolism in pre-diabetes [27]. Likewise, increased serum ferritin has been reported in relation to altered levels of VLDL-TG as well as HDL- and LDL-cholesterol [28,29], while serum ferritin is also increasingly recognized as marker for metabolic disease, in particular T2DM [6,7]. In line with this notion, we observed positive associations of serum ferritin with BCAAs and related intermediates which were shown to predict incident T2DM or fatty liver disease in numerous population-based studies [30,31,32]. Notably, the presented findings with respect to serum ferritin and plasma metabolites in women remained significant even after adjustment for fasting glucose (Figure S4). One previous study has provided first indications of a metabolic pathway that can lead to impaired glucose metabolism in case of elevated ferritin concentrations. The authors speculated about an implication *via* the metabolic pathways of the amino acids sarcosine and citrulline [33]. However, the larger metabolite coverage of our approach further augments their observation to BCAA catabolites but it remains elusive if those observational results reflect causal mechanisms or are surrogates for tissue-specific pathways. Nevertheless, the association between serum ferritin, metabolites and T2DM might provide a link to the adverse effect of red meat consumption as recently shown by Wittenbecher et al. [15] and it is of interest to identify the underlying mechanism in more detail, in particular given the strong sex-discrepancy in the present study (compare also Figure 2).

Not at least, serum ferritin is a well-known acute phase protein and hence a number of associated metabolites were overlapping with our previous metabolomics study on inflammatory parameters, comprising for example, acylcarnitines species [23]. Consistently, women in our study population appear to have subclinical but elevated levels of markers of inflammation *per se* (Table 1). 

In summary, the profile of associated metabolites with serum ferritin aligned with metabolomics signatures of incident type 2 diabetes and insulin resistance [34,35,36]. 

### 4.3. Haemoglobin and Lipoprotein Metabolism in Males

Apart from heme degradation, haemoglobin levels were positively associated with large VLDL-particles (VLDL_1_ and VLDL_2_) in men only, similar to serum ferritin. Large VLDL-particles are a primary secretion product of the liver and serve as carrier for newly synthesized TG, cholesterol and phospholipids to peripheral tissues. A possible link might be provided by the mutual positive association with plasma cortisol. Cortisol is known to stimulate hepatic glucose and lipid metabolism, including increased lipogenesis, VLDL assembly and cholesterol levels especially in response to stress [37,38,39]. Elevated cortisol levels are signs of pronounced chronic stress, as well as predictors of metabolic changes and are associated with diabetes and cardiovascular diseases [37,40,41]. Even in healthy men, both cortisol and haemoglobin appear to reactively increase in stressful situations [42]. However, the physiological link between cortisol and haemoglobin concentrations remains to be established.

### 4.4. Serum Transferrin and Lipoprotein Metabolism

Opposing to the male situation, serum transferrin was the strongest trait with respect to lipoprotein metabolism in females. However, while including large VLDL and IDL particles the associations were weaker as those seen with ferritin and haemoglobin among men and one might propose that transferrin is in women the more suitable iron-related marker to reveal iron-associated changes in lipoprotein metabolism. However, the underlying mechanism remains unclear. Of note, strong positive associations to transferrin levels were observed for small dense HDL_4_ particle measures in males as well as in females. Strikingly, Vaisar et al. have reported that HDL lipoprotein carry numerous proteins and peptides as investigated by shotgun proteomics combining LC-MS and biochemical analyses. Among several acute-phase proteins and complement-regulatory proteins reported in the fraction of small dense HDL particles also transferrin was found [43] which aligns with the report by McPherson [44] and is likely the underlying reason for our observations. 

### 4.5. Potential Markers to Predict Iron Depletion

Overall, women are more iron-depleted compared to men. This might be observed in changes in lipid metabolism related to subclinical iron depletion. In women, transferrin was inversely associated with some PC-species, while transferrin was positively associated with VLDL measures. It is well-known that PCs are necessary for assembly and secretion of VLDL lipoprotein particles from the liver. Notably, in iron deficiency anaemia VLDL levels have been reported to be elevated which was reversible by oral iron intake [45,46]. Increased consumption of PC species to assemble VLDL particles may account for the inverse association observed for transferrin implicating subclinical iron depletion. Likewise the positive association of PC species to ferritin may be interpreted in the same manner. As ferritin is reduced in iron depletion, PC species are lowered similarly due to increased assembly of VLDL particles. Yet, ferritin was not associated with VLDL measures in women. Reasons for this remain elusive but might be due to the absence of overt iron deficiency in the present study.

Our classification approach for possible novel markers related to iron status revealed in part expected characteristics like age or eGFR and was, consistent with the linear regression analyses, highly sex-specific. Some of the effects which are likely represented by the chosen markers, like free fatty acids, were already discussed in preceding sections. However, gamma-glutamylphenylalanine was the sole marker consistent for men and women and was further not obvious in linear regression analyses. Briefly, gamma-glutamylpeptides are formed as part of the gamma-glutamyl cycle for the restoration of the essential intracellular antioxidant glutathione (GSH) and consistently plasma concentrations of such dipetides have been shown to indicate increased (hepatic) oxidative stress [47]. Excess hepatic iron overload is a well-known cause of increased oxidative stress and therefore increased plasma concentrations of plasma gamma-glutamylpeptides likely reflect adverse consequences of iron rather than indicating iron depletion. Notably, the whole signature compiled for male participants showed strong alignment with the metabolomics profile of hepatic steatosis or hepatic damage [21] which might be an indication that those seem to suffer from iron overload rather than depletion, among others, resulting in impaired liver function as indicated by elevated serum ALT activities. To conclude, the compiled signatures comprise several aspects of whole body response to shifts in iron availability but other confounding factors might hamper their implementation as complementary markers for iron status.

### 4.6. A Short Note on the Strong Sex—Differences in Iron Metabolism

The most obvious female-specific determinants of iron homeostasis are menstruation, pregnancy and menopause [48,49]. Menstruation leads to lower iron availability due to an average blood loss of 40 mL per cycle [48], with variations from 5 to 26 mg iron lost per cycle [50]. During menopause, women suffer from both, lower iron values due to malabsorption [51] as well as from an iron overload [52] through a decreasing menstrual period due to lower oestrogen concentrations [53]. At least within the present study, menopause had only a minor modifying effect on associations between iron parameters and plasma metabolites. However, with respect to rhythmic changes during the menstrual cycle mutually affecting iron parameters and plasma metabolites we can only speculate. Plasma lipid concentrations vary depending on the cycle phase due to fluctuating oestrogen levels [54,55,56]. Thus, HDL-cholesterol appears to have its maximum around the ovulation, while LDL-cholesterol is highest in the follicular phase [55]. Similarly ferritin levels fluctuate across the menstrual cycle and hence introduce possibly a common variation of both parameters causing statistical correlation but not necessarily a causative relation. Unfortunately, data on the time point in the menstruation cycle of our female participants was not available and hence we could not rule out such residual confounding. Therefore, in order to analyse this more precisely, metabolome analyses, which can be precisely assigned to the individual cycle phases, should be carried out. 

### 4.7. Myoglobin Reflects Male Muscle Metabolism

With the exception of creatinine levels associations with myoglobin concentrations were only seen in males. Clinically, myoglobin is not a classical biomarker of iron metabolism but we included it in the present study as it is one of the most important iron containing enzymes. In particular, the inverse association with creatine and the positive association with creatinine are a hint towards altered energy metabolism in skeletal muscle as those reflect shifts in phosphocreatine utilization for the recycling of adenosine diphosphate to generate adenosine triphosphate. In line with this assumption, myoglobin is usually not present in plasma and its occurrence is due to muscle injury which perfectly fits with the shift in the creatine to creatinine ratio described. Another plausible explanation might arise from the implication of kidney function. This is highlighted in particular by a positive association with pseudouridine, a newly discovered metabolomic marker of kidney function [57]. Even if we accounted in linear regression analysis for the eGFR based on cystatin C, having the clear advantage to be far less affected by muscle mass compared to creatinine-based procedures, this might be not sufficient to truly rule out any influence. 

### 4.8. Strengths and Limitations

The strength of our study is reflected in the population size and the extensive metabolic profiling in combination with relevant circulating surrogates of iron metabolism in a healthy population. Nonetheless, the cross-sectional design of our study has limitations. Although associations are clearly visible, we can only speculate about causality. Further, it should be noted that we cannot rule out possible hidden factors, for example, the menstrual cycle, which might mediate some of the presented observations. A further drawback is the missing ability of detailed data on the nutritional behaviour of our participants.

## 5. Conclusions

The present study shows for the first time a comprehensive metabolic profile of important members in iron metabolism among a large population of apparently healthy volunteers. Our results provide hypothesis for molecular links to important (cardio-)metabolic disease, in particular through associations with highly resolved lipoprotein measures or BCAA intermediates with serum ferritin being the strongest biomarker. Further studies are needed to reveal if the sex-specific profile associated with serum ferritin might provide the link to a sexual-dimorphism of metabolic disease through metabolic maladaptation.

## Figures and Tables

**Table 1:** General characteristics of the study population.

Characteristics	Male (*N* = 409)	Female (*N* = 411)	*p* *
Age, years	50 (40; 61)	54 (44; 62)	0.93
Smoking, %			<0.01
Never	30.6	51.8	
Former	45.9	28.7	
Current	23.5	19.5	
Waist Circumference, cm	94 (87; 102)	83 (76; 91)	<0.01
Ferritin, µg/L	149 (86; 255)	60 (28; 104)	<0.01
Transferrin, g/L	2.5 (2.2; 2.7)	2.5 (2.3; 2.8)	<0.01
Myoglobin, µg/L	58 (50; 71)	44 (37; 54)	<0.01
Haemoglobin, mmol/L	9.1 (8.8; 9.5)	8.3 (7.9; 8.6)	<0.01
eGFR, mL/min/1.73 m²	116 (108; 126)	110 (101; 118)	<0.01
ALT, µkatal/L	0.47 (0.35; 0.65)	0.31 (0.25; 0.44)	<0.01
hsCRP, mg/L	0.99 (0.56; 1,83)	1.16 (0.62; 2.45)	<0.01
Fibrinogen, g/L	2.8 (2.3; 3.3)	3.1 (2.6; 3.5)	<0.01
Glucose, mmol/L	5.4 (5.1;5.8)	5.2 (4.9; 5.6)	<0.01

Data are expressed as median (25th; 75th percentile). eGFR = estimated glomerular filtration rate; ALT = alanine aminotransferase; * *p*-value resulting from Wilcoxon-rank-sum test for continuous and χ²-test for categorical data was used for comparison.

## Supplementary Materials

The following are available online at http://www.mdpi.com/2072-6643/10/11/1800/s1, Figure S1: Comparison of beta-estimates from linear regression analyses for all iron traits after separating women in pre- and postmenopausal women. Figure S2: Final results from classification analyses using random forests in a two-stage cross-validation scheme for women (A) and men (B). Figure S3: Association heatmap of lipid species significantly associated with at least one trait under investigation. Figure S4: Comparison of *p*-values from linear regression models. Table S1: Significantly associated plasma metabolites with at least one of the surrogates of iron metabolism.
