# Supplementary material for: Molecular Fingerprints of Iron Parameters among a Population-Based Sample
Source: Nutrients. 2018 Nov 19;10(11):1800. doi: 10.3390/nu10111800 (PMC6266982; doi:10.3390/nu10111800)
Supplement: Supplementary file 1 [file nutrients-10-01800-s001.zip › 1-nutrients-382033-supplementary-final/Supplement-1.docx]

SUPPLEMENTARY FIGURES


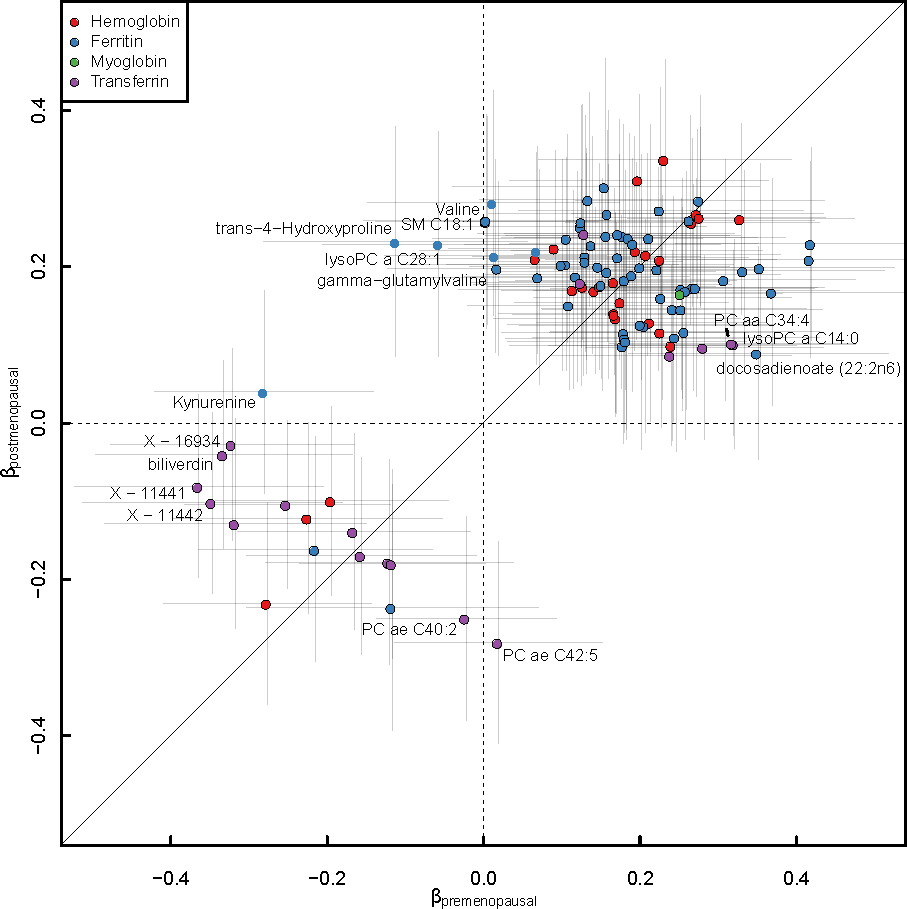


**Figure S1** Comparison of beta-estimates from linear regression analyses for all iron traits after separating women in pre- and postmenopausal women. Metabolites were only depicted if they were significantly associated with at least one trait (false discovery rate <5%) either in all women (framed circles) or among one of the subpopulations. Confidence intervals are provided as an equivalent to nominal testing. Metabolites were marked with names in case a nominal significant modifying effect of menopause on the association was apparent from statistical analyses. Prior plotting, estimates were rescaled to reflect a one standard deviation increase in every iron trait.

**
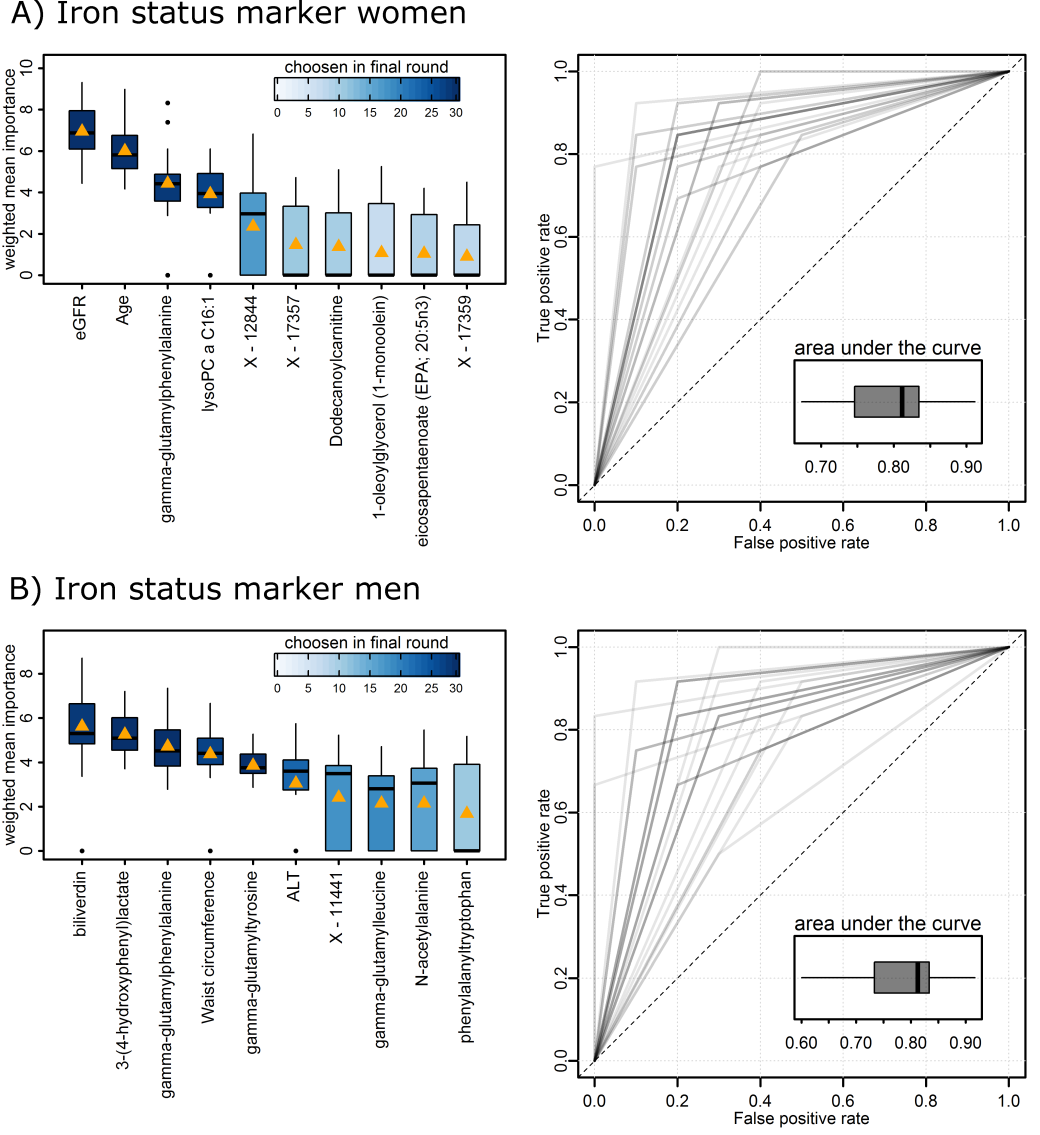
**

**Figure S2** Final results from classification analyses using random forests in a two-stage cross-validation scheme for women (A) and men (B). Left panel: Ten most important metabolites ranked by a weighted (area under the curve) mean Gini index (orange triangle). Boxplots indicate distribution across 30 outer validation runs. Right panel: Receiver operating characteristic (ROC) curve and boxplot of the area under the curve from 30 validation runs. Overlapping ROCs are displayed by darker shades


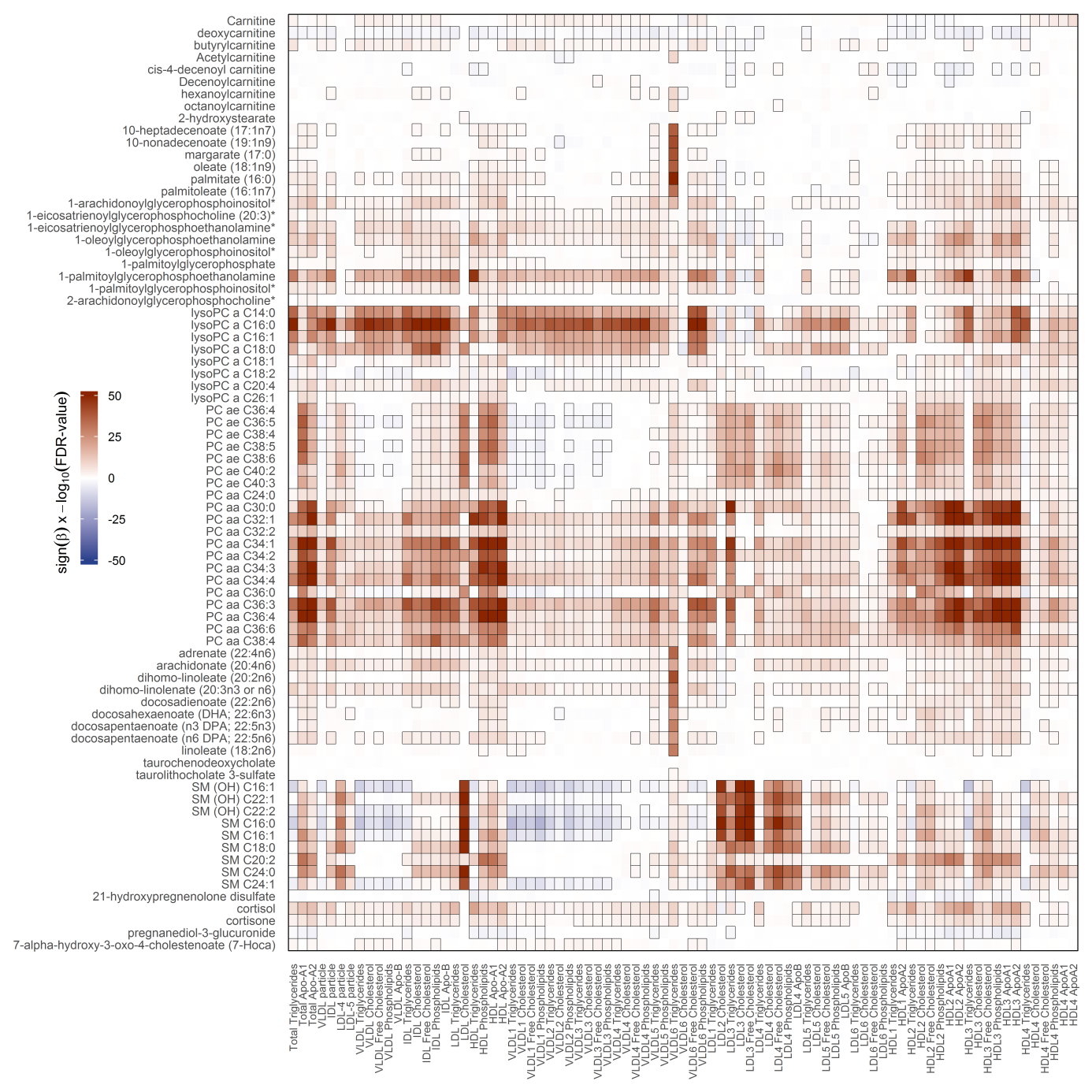


**Figure S3** Association heatmap of lipid species significantly associated with at least one trait under investigation. Colors indicate corrected p-values (controlling the false discovery rate [FDR]) from linear regression analyses using a lipoprotein subclass as exposure and one metabolite as outcome adjusting for age, sex and body mass index. Orange shadings indicate positive associations whereas blue indicates the opposite direction.


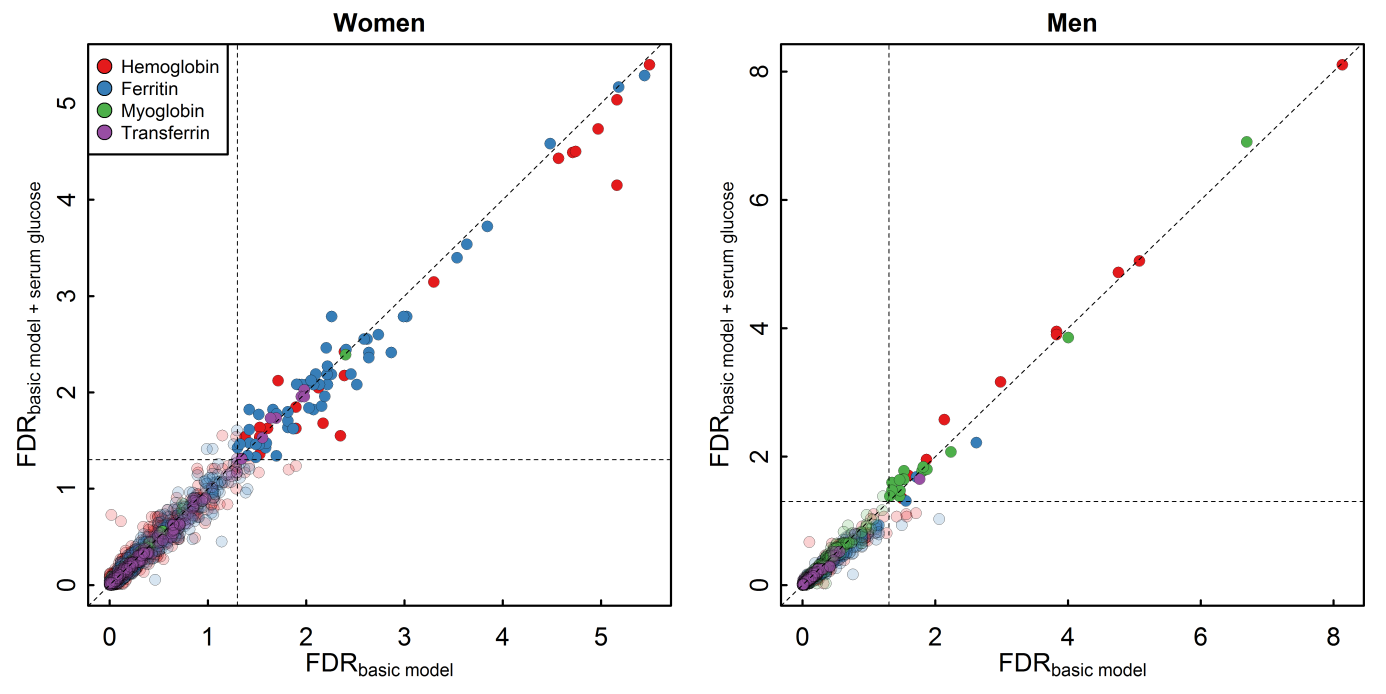


**Figure S4** Comparison of P-values from linear regression models using either the basic model (adjusting for age, waist circumference, smoking behaviour, estimated glomerular filtration and, serum alanine aminotransferase activities) and after further adjustment for fasting glucose measurements. *p*-values were corrected for multiple testing by controlling the false discovery rate (FDR) at 5% (darker colours).
